# Air pollution alters *Staphylococcus aureus* and *Streptococcus pneumoniae* biofilms, antibiotic tolerance and colonisation

**DOI:** 10.1111/1462-2920.13686

**Published:** 2017-02-28

**Authors:** Shane. J. K. Hussey, Joanne Purves, Natalie Allcock, Vitor E. Fernandes, Paul S. Monks, Julian M. Ketley, Peter W. Andrew, Julie A. Morrissey

**Affiliations:** ^1^ Department of Genetics, Adrian Building University of Leicester, University Road Leicester LE1 7RH Leicestershire, UK; ^2^ Centre for Core Biotechnology Services, Adrian Building University of Leicester, University Road Leicester LE1 7RH Leicestershire, UK; ^3^ Department of Infection Immunity and Inflammation, Medical Sciences Building, University of Leicester, University Road Leicester LE1 9HN Leicestershire, UK; ^4^ Department of Chemistry University of Leicester, University Road Leicester LE1 7RH Leicestershire, UK

## Abstract

Air pollution is the world's largest single environmental health risk (WHO). Particulate matter such as black carbon is one of the main components of air pollution. The effects of particulate matter on human health are well established however the effects on bacteria, organisms central to ecosystems in humans and in the natural environment, are poorly understood. We report here for the first time that black carbon drastically changes the development of bacterial biofilms, key aspects of bacterial colonisation and survival. Our data show that exposure to black carbon induces structural, compositional and functional changes in the biofilms of both *S. pneumoniae* and *S. aureus*. Importantly, the tolerance of the biofilms to multiple antibiotics and proteolytic degradation is significantly affected. Additionally, our results show that black carbon impacts bacterial colonisation *in vivo*. In a mouse nasopharyngeal colonisation model, black carbon caused *S. pneumoniae* to spread from the nasopharynx to the lungs, which is essential for subsequent infection. Therefore our study highlights that air pollution has a significant effect on bacteria that has been largely overlooked. Consequently these findings have important implications concerning the impact of air pollution on human health and bacterial ecosystems worldwide.

## Introduction

Particulate matter (PM), a major component of air pollution (Kelly and Fussell, 2011; Kelly and Fussell, 2012), has a detrimental impact on both human and environmental health (Janssen *et al*., 2012; Kelly and Fussell, 2012; Thurston and Lippmann, 2015; Xu *et al*., 2016). Current WHO guidelines set limits for both the 24‐hour and annual mean concentrations of different size fractions of PM. PM_10_, that is, particulate matter with an aerodynamic diameter of less than 10 µm, should be kept below a 20 µg/m^3^ annual mean, and a 50 µg/m^3^ 24‐hour mean. Stricter limits are set for PM_2.5_ as this size fraction is associated with more damaging health effects, and should be kept below an annual mean of 10 µg/m^3^, and 25 µg/m^3^ over a 24 hour period. However despite WHO and EU legislation to reduce air pollution, PM levels exceed these recommended guidelines (WHO, 2006, 2016), particularly in industrialised regions (Janssen *et al*., 2012; Wang *et al*., 2014).

PM and other air pollutants spread in the atmosphere and cross national boundaries, settling on plants and soils, as well as contaminating fresh and marine water (Forbes *et al*., 2006; Cattaneo *et al*., 2010). PM therefore has the potential to affect multiple essential ecosystems globally, as well as having a known major impact on human health and morbidity. PM exposure causes increased respiratory and cardiovascular disease (Faustini *et al*., 2012; Shah *et al*., 2015; Thurston and Lippmann, 2015; Costello *et al*., 2016), and are strongly associated with increased acute respiratory infections, including pneumonia (Brugha and Grigg, 2014; MacIntyre, 2014; Qiu *et al*., 2014; Chang *et al*., 2015; Xu *et al*., 2016). Indeed, air pollution is responsible for an eighth of all global deaths per year (WHO, 2014).

Many studies have shown that inhalation of PM causes host tissue damage, inflammation, oxidative stress and alteration in cardiovascular functioning, as well as significantly impacting the immune response by impairing macrophage function (Host *et al*., 2007; Lundborg *et al*., 2007; Kelly and Fussell, 2011, 2012; Heal *et al*., 2012; Rylance *et al*., 2015). However, these host‐focused studies do not fully account for all the observations of PM‐related diseases in humans. Importantly, there is a major omission in our understanding of the impact of PM because there have been no studies on the direct impact of PM on the behaviour of bacteria. This is surprising considering that bacteria are directly responsible for respiratory infections, and play a key role in the diversity and functioning of the normal microbiome, which is crucial for maintaining the health of the host. Therefore it is essential to further understand the role bacteria play in the detrimental impacts of air pollution.

A major component of PM is black carbon (BC), a by‐product of fossil fuel combustion. In developed countries diesel exhaust fumes are the major source of BC, whereas in the developing world BC mostly arises from indoor burning of biomass for heat and fuel. BC is a chemically and biologically active pollutant that can generate oxidative stress, induce inflammation, and be mutagenic (Janssen *et al*., 2012; Butterfield *et al*., 2015). In 2014, black carbon (BC), a major component of PM, levels ranged from 1 to 7 µg/m^3^ across the UK, and the country‐wide average was 1.6 µg/m^3^ (Butterfield *et al*., 2015). In general, higher concentrations were recorded at the roadside in comparison to other urban environments. However, Europe and North America only account for about 13% of global BC emissions, whereas developing countries are responsible for ∼80%, with the biggest global contributors to BC being China and India (USEPA, 2012; Ni *et al*., 2014; Wang *et al*., 2014). BC exposure is strongly implicated in predisposition to respiratory infectious disease, which is particularly damaging to children under 5 years old (Janssen *et al*., 2012; Brugha and Grigg, 2014).

To address whether bacteria are an unexplained mechanism for BC induced morbidity, we investigated the impact of BC on two model bacterial species; *Streptococcus pneumoniae* and *Staphylococcus aureus*. Both are important respiratory tract commensals intermittently carried by a large section of the population without signs of disease as part of the normal microbiome. However they are also globally important human pathogens; *S. pneumoniae* is the leading bacterial cause of pneumonia, and *S. aureus* is a significant cause of respiratory and skin and tissue disease (Wertheim *et al*., 2005; Edwards *et al*., 2012; Shak *et al*., 2013).

We report here that BC significantly affects the behavior of *S. pneumoniae* and *S. aureus*. Our data show that BC impacts biofilm formation, an essential aspect of bacterial colonisation and environmental survival. Exposure to BC induced significant changes in *S. pneumoniae* and *S. aureus* biofilm structure, composition and function. Importantly, BC differentially altered the tolerance of biofilms to proteolytic degradation and multiple antibiotics, increasing *S. pneumoniae* survival against penicillin, the front line treatment of bacterial pneumonia. Furthermore this work shows that BC does indeed impact bacterial colonization *in vivo*. In a murine colonisation model, black carbon induced *S. pneumoniae* to spread from the nasopharynx to the lungs, which is a prerequisite for invasive disease in a susceptible host. Therefore, if extrapolated these data show, for the first time, that air pollution could have a significant effect on human bacterial infection that has been largely overlooked.

## Results

### Black carbon alters S. pneumoniae and S. aureus biofilm structure

To investigate our hypothesis that air pollution alters bacterial colonisation and hence impacts environmental survival, the impact of black carbon on biofilm formation was determined. Bacteria within biofilms are highly protected against environmental stresses; including metals, protease degradation, the host immune response and antibiotics (Domenech *et al*., 2012; Marks *et al*., 2012; De la Fuente‐Nunez *et al*., 2013; Nicholson *et al*., 2013). Therefore, biofilms are an important facet of colonisation and can act as a reservoir for infection, dispersing bacteria to spread to other sites (Marks *et al*., 2013).

**Figure 1:**
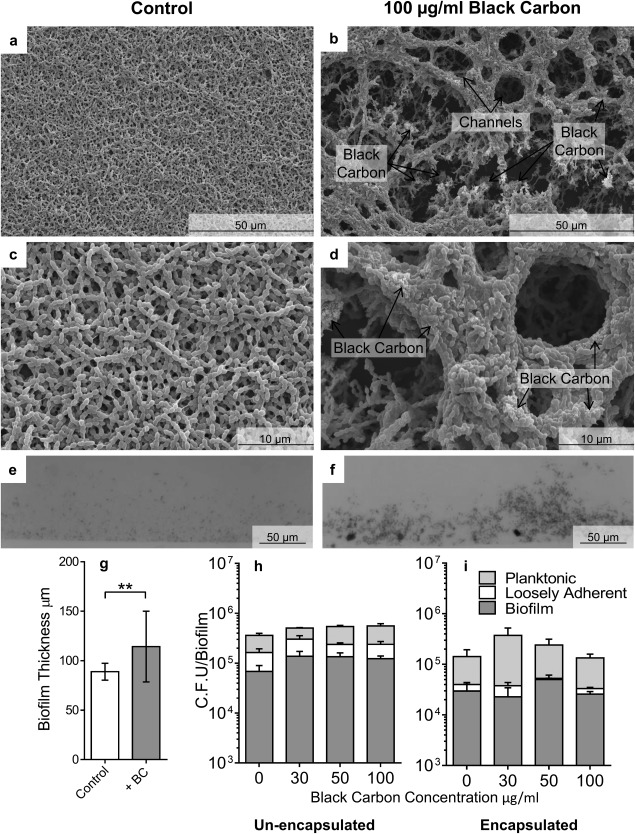
The effect of black carbon on *S. pneumoniae* biofilm structure. Biofilms of *S. pneumoniae* PR201 (a–h) and D39 (i) were cultured in the presence or absence of 30–100 μg/ml BC. Biofilms were imaged by Scanning Electron Microscopy (SEM) at increasing resolutions (a–d). Light microscopy was used to quantify biofilm thickness (e.g. *n* = 18). Images are representative of the entire biofilm structure. Viable bacterial cells were measured by sequential removal and quantification of planktonic, loosely‐adhered, and biofilm bacteria (h, i, *n* = 4). Error bars represent ± 1 SEM. Significance was determined by *t*‐tests (g) or ANOVA (h, i). **=*p* ≤ 0.01.

**Figure 2:**
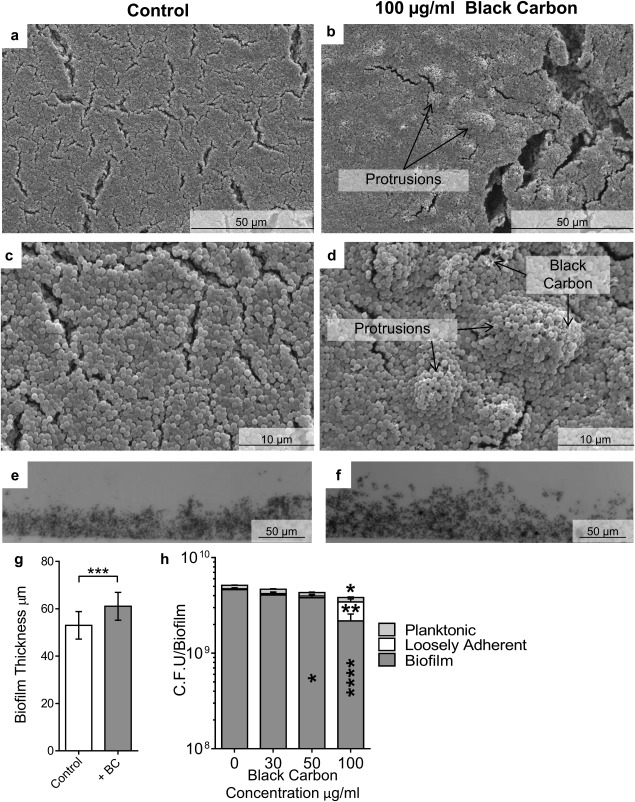
The effect of black carbon on *S. aureus* biofilm structure. Biofilms of *S. aureus* SH1000 were cultured in the presence or absence of 30–100 μg/ml BC. Biofilms were imaged by scanning electron microscopy at increasing resolution (a–d) and light microscopy was used to quantify biofilm thickness (e.g., *n* = 18). Images are representative of the entire biofilm structure. Viable bacterial cells were measured by sequential removal and quantification of planktonic, loosely‐adhered, and biofilm bacteria (h, *n* = 4). Error bars represent ± 1 SEM. Significance was determined by *t*‐tests (g) or ANOVA (h) *=*p* ≤ 0.05, **=*p* ≤ 0.01, ***=*p* ≤ 0.001, ****=*p* ≤ 0.0001.

To investigate the impact of BC on bacterial biofilms, *S. pneumoniae* strains D39 and PR201, and *S. aureus* strains SH1000, Newman, and USA300, were exposed to 30–100 µg/ml BC during biofilm formation. BC concentrations were based on previous research into the effects of BC on the host, as well as research into the effects of other pollutants on bacteria (Tellabati *et al*., 2010; Vesterdal *et al*., 2012; Suraju *et al*., 2015). These concentrations do not represent actual atmospheric BC concentrations in the UK which range from 1 to 7 µg/m^3^ (Butterfield *et al*., 2015) because atmospheric BC is inhaled over time, with constant deposition within the respiratory tract, therefore high concentration suspensions are typically used to model this long‐term exposure. Both encapsulated (D39) and non‐capsulated (PR201) pneumococci were used to determine if the presence of a capsule altered the response to BC. In addition, encapsulated *S. pneumoniae* form poor biofilms *in vitro* therefore investigations using advanced imaging could only be completed with the closely‐related non‐capsulated isolate. The impact on biofilm formation was assessed by advanced microscopy and by enumeration of viable biofilm and planktonic bacteria, as well as those cells loosely adhered to the biofilm. Interestingly, the data show that BC drastically alters bacterial biofilm formation (Figs 1 and 2) and notably, a striking and differential impact was observed between the biofilm structures of *S. pneumoniae* and *S. aureus*.

BC caused a dramatic alteration in the architecture of non‐capsulated *S. pneumoniae* PR201 biofilms, resulting in a structure with complex protrusions and channels (Fig. 1b and d). This is in contrast to *S. pneumoniae* biofilms formed in the absence of BC, which were relatively flat structures with a low surface area, limiting environmental contact (Fig. 1a and c). BC‐induced biofilms were also found to be significantly thicker and more irregular than control biofilms (Fig. 1e–g, *p* ≤ 0.01). TEM showed that the pneumococcal cells within the biofilm were closely associated with the BC particles (Supporting Information Fig. S1a and b), a feature also observed by SEM (Fig. 1b and d). This proximity to the BC had no obvious impact on the *S. pneumoniae* cell shape, but in the presence of BC there appeared to be a higher proportion of dividing cells and fewer ‘ghost’ dead cells, correlating with an increase in the number of viable *S. pneumoniae* found in the biofilm (Fig. 1h); although BC had no significant effect on total cell viability of either *S. pneumoniae* strain investigated.

The BC‐induced complex architecture and increased thickness of the biofilm did not affect biofilm integrity because there was no change in the proportion of loosely associated and planktonic *S. pneumoniae* cells compared with biofilm cells. This was true for encapsulated and non‐capsulated *S. pneumoniae* (Fig. 1h and i). Interestingly, *S. pneumoniae* biofilms formed in the presence of quartz, a particle of similar size to BC but chemically inert, did not induce any changes in biofilm structure (Supporting Information Fig. S2c and d). This shows that the effect of BC on biofilm formation is not merely due to the physical presence of exogenous particles acting as a scaffold for the biofilm rather it demonstrates that BC induces a biological effect on the bacteria.

BC also altered *S. aureus* biofilm formation, but different effects were observed. Similar to *S. pneumoniae*, BC significantly increased the average thickness of *S. aureus* biofilms (Fig. 2e–g; *p* ≤ 0.001; Supporting Information Fig. S3e–g; *p* ≤ 0.001). Additionally, the biofilm structure was altered in that BC induced development of bulky masses that protruded from the normally smooth surface of the biofilms, although the structure observed was not as complex as that of *S. pneumoniae* BC‐biofilms (Fig. 2b, d, f, and g, Supporting Information Fig. S3b, d and f, and S4b and d). In contrast to *S. pneumoniae*, BC exposure altered the integrity of the *S. aureus* biofilm. In the absence of BC, *S. aureus* biofilms were formed with only a small proportion of the total number of cells loosely‐adhered to the biofilm (Fig. 2h; Supporting Information Fig. S3h and S4e). In the presence of BC, however, there was a significant decrease in the number of biofilm cells (Fig. 2h; *p* ≤ 0.0001, Supporting Information Fig. S3h *p* ≤ 0.0001), and a significant increase in the loosely associated cells (Fig. 2h; *p* ≤ 0.001), showing that biofilm structural integrity had been altered.

Unexpectedly, unlike *S. pneumoniae*, BC was found to be detrimental to *S. aureus* strains SH1000 and Newman, because exposure to BC significantly decreased (*p* ≤ 0.05) the total number of viable *S. aureus* cells compared with the control (Fig. 2h, Supporting Information Fig. S3h). Interestingly however, BC did not significantly affect the viability of the methicillin resistant *S. aureus* (MRSA) strain USA300 (Supporting Information Fig. S4e), demonstrating that the effect of BC differs between *S. aureus* strains. TEM analysis did not reveal any significant effect of BC on the structure of the *S. aureus* cells (Supporting Information Fig. S1c–f). Together our data show that BC changes bacterial biofilm formation and structure by two model bacteria, but importantly, there is intra‐ and inter‐species variation in the impact of BC.

### Black carbon induces changes in proteolytic degradation of the biofilm

To investigate whether BC affects biofilm function, the biofilms were treated with proteinase K to determine the impact on the tolerance to proteolytic degradation (Nicholson *et al*., 2013). Non‐BC exposed biofilms of *S. aureus* Newman and *S. aureus* USA300 were significantly disrupted by treatment with proteinase K, showing that these biofilms are mainly proteinaceous in composition (Table 1 *p* ≤; 0.01). In contrast, *S. aureus* SH1000 and the *S. pneumoniae* PR201 control biofilms were not significantly disrupted by proteinase K treatment, suggesting that these biofilms contained less protein (Table 1).

**Table 1:** Biofilm degradation by Proteinase K. Viability of pre‐formed biofilms in the presence (+BC) or absence (‐BC) of black carbon, after incubation with 100 µg/ml Proteinase K for 2 h. *n* = 3. The *p* value was determined by ANOVA ** *p* ≤ 0.01, **** *p* ≤ 0.0001. ns = not significant. Percentage degradation describes data, but was not used for statistical analysis.

	‐ BC				+ BC			
	‐PK	+PK	p value	% Degradation	‐PK	+PK	p value	% Degradation
Newman	2.20 × 10^9^	1.26 × 10^9^	**	**43**	9.20 × 10^8^	7.57 × 10^8^	ns	**18**
USA300	2.83 × 10^9^	1.58 × 10^9^	**	**44**	1.48 × 10^9^	9.23 × 10^8^	ns	**38**
SH1000	4.52 × 10^9^	3.67 × 10^9^	ns	**19**	4.17 × 10^9^	2.57 × 10^9^	**	**38**
*S. pneumoniae*	5.15 × 10^5^	3.17 × 10^5^	ns	**38**	5.87 × 10^5^	1.23 × 10^5^	**	**79**

Interestingly BC exposure altered the sensitivity of all biofilms to proteinase K. BC‐biofilms of *S. aureus* Newman and USA300 became less sensitive to proteinase K degradation, whereas the *S. aureus* SH1000 and *S. pneumoniae* BC‐exposed biofilms became considerably more degraded by proteinase K (Table 1). These results suggest that BC has a notable effect on the protein component of the biofilms, reducing protein content of the S*. aureus* Newman and USA300 biofilms but increasing that of *S. aureus* SH1000 and *S. pneumoniae*.

### Black carbon alters the tolerance of biofilms to multiple antibiotics

Bacterial biofilms are known to be highly resistant to antibiotics therefore the effect of BC on tolerance of the biofilms to antibiotics was assessed. Preformed biofilms grown in the presence or absence of 100 µg/ml BC were washed and incubated with or without antibiotics in growth medium for 3 h. 100 µg/ml BC results in major structural changes and therefore this concentration is optimal to test if BC induces functional changes. After incubation, bacterial viability was assessed. The percentage survival of bacteria exposed to antibiotics, compared with non‐exposed controls, was then calculated. *S. aureus* and *S. pneumoniae* were exposed to the β‐lactams oxacillin and penicillin G, respectively, which inhibit cell wall synthesis. *S. aureus* biofilms were also exposed to daptomycin, a lipopeptide which disrupts the cell membrane, and tetracycline, which inhibits intracellular protein synthesis.

**Figure 3:**
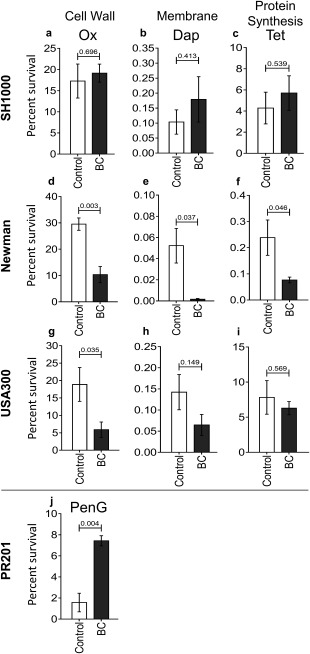
The effect of black carbon (BC) on biofilm tolerance to antibiotics. Biofilms of *S. aureus* SH1000 (a–c), Newman (d–f), and USA300 (g–i), and *S. pneumoniae* PR201 (j) were cultured in the presence or absence of 100 μg/ml BC for 24 h. Planktonic and loosely‐adhered bacteria were then removed and biofilms were either incubated with 3 ml of an antibiotic‐containing medium or control medium for 3 h at 37°C in 5% (v/v) CO_2_. For *S. aureus*, oxacillin (50 mg/ml in BHI + 2% (w/v) NaCl), daptomycin (256 µg/ml in BHI + 50 µg/ml CaCl_2_) and tetracycline (512 µg/ml in BHI) were used. For *S. pneumoniae* PR201, penicillin G (1 µg/ml) was used in BHI. Planktonic and loosely‐adhered cells were then removed and biofilm bacteria quantified. *n* ≥ 3. Error bars represent ± 1 SEM. Significance was determined by unpaired *t*‐tests.

It is noteworthy that the concentration of each antibiotic required to markedly reduce the viability of bacteria within control *S. aureus* biofilms was considerably higher than the minimum inhibitory concentration (MIC) needed for planktonic bacteria (Fig. 3). Planktonic *S. aureus* cells are sensitive to ∼2 μg/ml oxacillin, 1 μg/ml daptomycin, and 2 μg/ml tetracycline (EUCAST, 2015), whereas 50 mg/ml oxacillin, 256 μg/ml daptomycin and 512 μg/ml tetracycline were required to reduce the viability of *S. aureus* biofilms. None of these antibiotic concentrations resulted in total bacterial eradication in control biofilms, clearly demonstrating that biofilms have increased antibiotic tolerance compared with planktonic cells.

Our data show that BC has a differential effect on bacterial biofilm tolerance to antibiotics irrespective of the mode of action. *S. aureus* Newman and the MRSA USA300 biofilms formed in the presence of BC showed significant decreases in tolerance to oxacillin (Fig. 3d; *p* = 0.03 and Fig. 3g; *p* = 0.035 respectively). BC‐biofilms of *S. aureus* Newman also showed significant decreases in tolerance to daptomycin and tetracycline (Fig. 3e; *p* = 0.037, and Fig. 3f; *p* = 0.046. In contrast, BC had no significant effect on percentage survival of *S. aureus* SH1000 biofilms exposed to any of the test antibiotics (Fig. 3a–c), instead there was a reproducible but non‐significant increase in survival of BC‐biofilms for all three antibiotics. This increase in SH1000 tolerance to oxacillin was significant, however, when biofilms were exposed to the antibiotic in PBS and not growth media (Supporting Information Fig. S5; *p* ≤ 0.01); *S. aureus* Newman and USA300 also still demonstrated the observed significant decrease in tolerance to oxacillin using this method (Supporting Information Fig. S5; *p* ≤ 0.05).Importantly, biofilms of *S. pneumoniae* formed in the presence of BC showed a significantly increased resistance to penicillin G, a front line antibiotic (Fig. 3j; *p* < 0.004). *S. pneumoniae* is the major cause of pneumonia worldwide therefore this has major implications for the treatment of this disease.

To allow for comparison of the antibiotic tolerance of different strains, data was presented as percentage survival (Fig. 3). It should be noted however that small percentage changes correlate to biologically relevant differences in bacterial CFUs. For example, the percentage survival of *S. aureus* Newman biofilms after treatment with daptomycin is 0.043% (Fig. 3e) in the control condition, and 0.002% for biofilms formed in the presence of BC. In the control condition, this corresponds to 3.26 × 10^9^ CFU/ml without antibiotic treatment and 1.41 × 10^6^ CFU/ml with daptomycin treatment, which represents a 3 log decrease. Whereas BC‐formed biofilms had a CFU/ml of 1.99 × 10^9^ in the absence of daptomycin treatment, and 4.12 × 10^4^ CFU/ml with daptomycin treatment, representing a 5 log decrease.

Together, our data show that BC affects the function of bacterial biofilms by altering tolerance to antibiotics with different modes of action. Additionally, our data show that there is an association between the tolerance of the biofilms to antibiotics and the level of proteolytic degradation. Biofilms of *S. aureus* Newman and USA300 formed with BC demonstrated decreased degradation by proteinase K in comparison to controls, and a decreased tolerance to antibiotics. In contrast, biofilms of *S. aureus* SH1000 and *S. pneumoniae* formed with BC both show increased degradation by proteinase K in comparison to controls, and reduced sensitivity to β‐lactam antibiotics. Therefore these data suggest an association between the overall biofilm protein composition and the protectivity of these biofilms against antibiotics.

### Black carbon promotes spread of bacteria to the lungs

A significant percentage of the population carries *S. pneumoniae* asymptomatically in the nasopharynx, and dissemination of *S. pneumoniae* from the nasopharynx to the lower respiratory tract is key to subsequent pneumococcal disease. Bacteria, including *S. pneumoniae*, can be introduced into the nasopharynx through inhalation of PM/bacterial aggregates (Cao *et al*., 2014). Therefore, to determine whether BC affects bacterial colonisation and subsequent dissemination, a *S. pneumoniae* murine colonisation model was used. Normally with this *in vivo* model, there is asymptomatic colonisation of the nasopharynx but no spread of bacteria into the lungs, or the bloodstream (Richards *et al*., 2010).

Mice were intranasally inoculated with (i) BC alone, (ii) non‐BC treated *S. pneumoniae*, (iii) *S. pneumoniae* with BC or (iv) saline as a control. Blood was taken and nasopharyngeal and bronchoalveolar lavages were performed to determine bacterial carriage at predetermined time‐points. A concentration of 7mg/ml BC was used because this does not produce any adverse host response and therefore any observed effect is due to the bacteria and not due to a significant effect on the host. The lungs, spleen, cervical lymph nodes and nasopharyngeal tissue were taken from separate mice for histological analysis.

**Figure 4:**
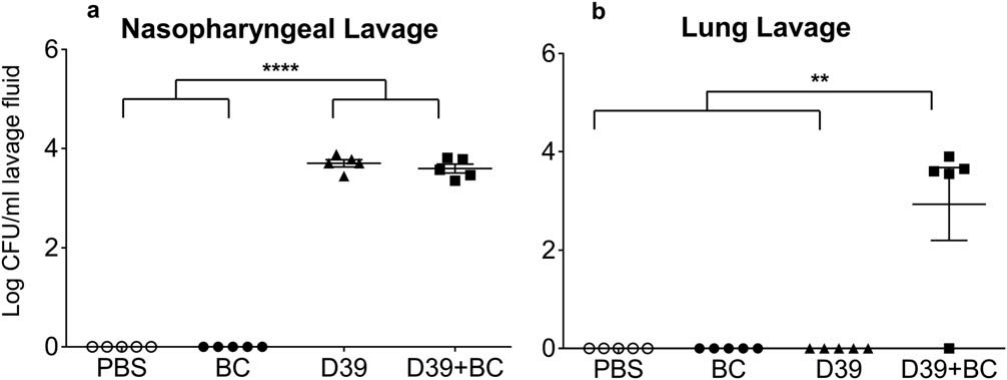
The effect of black carbon on respiratory tract colonisation. Female MF1 mice were intranasally inoculated with 15 µl of PBS, 7 mg/ml BC, 6.45 × 10^5^ CFU of *S. pneumoniae* D39, or a combination of BC and *S. pneumoniae* D39. At 7 days post‐inoculation, nasopharyngeal (a) and lung (b) lavages were performed and serial dilutions were plated out to determine bacterial load. No mice showed clinical signs of morbidity. *n* = 5 per group. Conditions were compared with individual *t*‐tests. Error bars represent ± 1 SEM. Statistical significance is denoted with * *p* ≤ 0.05.

Significantly, the data show that BC promotes the spread of *S. pneumoniae* from the nasopharynx to the lungs (Fig. 4). At 7 days post‐inoculation, there was the same level of colonisation in the upper respiratory tract of mice co‐exposed to *S. pneumoniae* and BC, and those infected with *S. pneumoniae* alone (Fig. 4a). In contrast, in the lungs, bacteria were only detected when the mice had been exposed to *S. pneumoniae* and BC (Fig 4b; *p* ≤ 0.01). No *S. pneumoniae* were found in the blood in any condition. Histological analysis of the nares, lungs, cervical lymph nodes and spleen of control and BC‐exposed mice did not reveal any overt signs of an inflammatory response nor were any BC particles detected at these sites (Supporting Information Figs S6 and S7). The mice exposed to BC and *S. pneumoniae* did not display any overt clinical signs of disease despite the observation of ∼3.68 Log10 CFU/ml in the bronchoalveolar lavages. The fact that *S. pneumoniae* is only detected in the lungs of mice exposed to BC shows that inoculation in the presence of BC promotes the dissemination of *S. pneumoniae* to the lower respiratory tract, increasing the opportunity for bacteria to cause invasive disease such as pneumonia.

## Discussion

In this study we show that a major component of air pollution, BC, significantly affects bacterial colonisation and biofilm formation, thereby demonstrating that a biological effect of air pollution has been overlooked. Consequently this work establishes a new paradigm; that the detrimental impact of particulate pollutants on human health is not only just due to direct effects on the host, but also involves the effect on bacterial behaviour in the host.

Our data provides strong evidence for the first time that BC alters bacterial colonisation. In the presence of BC, *S. pneumoniae* biofilms were considerably thicker and more complex than their control counterparts, as well as substantially more sensitive to proteolytic cleavage and importantly, more resistant to antibiotics. The biofilm structure induced by BC in this study is characteristic of the honey‐comb structure of pneumococcal biofilms only previously observed *in vivo* or on biotic surfaces *in vitro* (Marks *et al*., 2012). This suggests that BC acts as a novel signal to induce pneumococcal biofilm formation, promoting colonisation of the respiratory tract. The increased sensitivity of *S. pneumoniae* biofilms to proteolytic degradation is also interesting because *in vivo*, where *S. pneumoniae* will be exposed to degrading enzymes, it could result in increased dispersal of the biofilm and subsequent colonisation of *S. pneumoniae* at other body sites. The increase in antibiotic resistance of pneumococcal biofilms formed in the presence of BC is particularly worrying as this could affect the treatment of pneumococcal infections during exposure to PM.

BC also impacts the behavior of *S. aureus* by inducing thicker, more irregular biofilms with decreased structural integrity. These biofilm changes could also result in an increase in the detachment and dissemination of *S. aureus* from the biofilm *in vivo*. Interestingly BC appeared to inversely affect the sensitivity of the *S. aureus* biofilms to proteinase K and antibiotics. *S. aureus* Newman and USA300 BC‐formed biofilms demonstrated decreased sensitivity to proteinase K and increased antibiotic sensitivity compared with non‐exposed biofilms. In contrast, *S. aureus* SH1000 showed increased proteinase K sensitivity and decreased antibiotic sensitivity. These data suggest that BC exposure results in changes in biofilm composition that directly alter biofilm function. Additionally, these data suggest a novel link between total biofilm protein composition and tolerance to different classes of antibiotics. This association could be due to BC inducing specific proteins such as the penicillin binding proteins, proteins that change the surface charge, or efflux transporters, or a more general change in the overall protein composition. Further investigation is required to determine the specific mechanisms by which these changes occur as well as the impact on tolerance to other environmental stressors such as the immune response.

Our data demonstrate that BC also impacts colonisation *in vivo* and promotes the spread of *S. pneumoniae* from the nasopharynx to the lungs in a murine respiratory tract model. The determinants of the shift from asymptomatic colonisation to invasive disease have yet not been fully established. Our data identify a novel specific signal that induces S*. pneumoniae* to shift from colonisation of the nasopharynx to the lungs. These results suggest that BC induction of *S. pneumoniae* dissemination and subsequent colonisation of the lungs could be a key factor in how air pollutants cause increased lower respiratory tract infectious disease.

**Figure 5:**
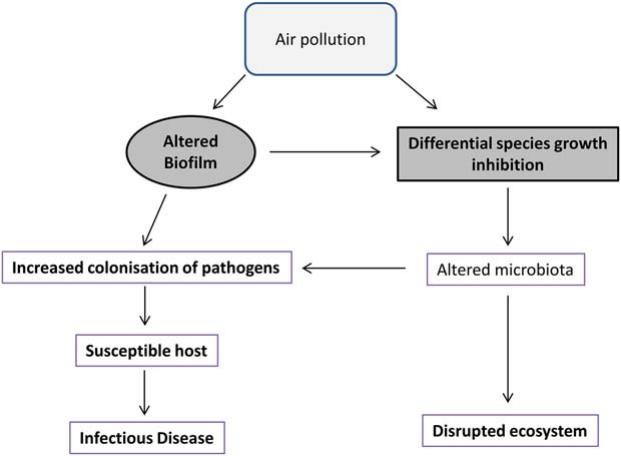
Proposed model of the impact of air pollution on bacterial infection and environmental ecosystems. [Colour figure can be viewed at http://wileyonlinelibrary.com]

The presence of *S. pneumoniae* in the lungs did not cause any clinical signs of disease nor did the bacteria induce any inflammatory changes. The lack of an inflammatory response could be due to BC particles altering the immune system, as has been previously observed, thereby reducing the capacity of the host immune system to mount a response to the bacteria (Zhou and Kobzik, 2007; Mushtaq *et al*., 2011; Chaudhuri *et al*., 2012; Mannucci *et al*., 2015; Rylance *et al*., 2015; Longhin *et al*., 2016). Alternatively, although BC increased the spread of bacteria to the lower respiratory tract (LRT) and thereby provided an increased potential to cause disease, this may only be realised in a susceptible host, such as the very young, or the elderly (Janssens and Krause, 2004; O'Brien *et al*., 2009), or further acute or chronic exposure to stress such as pollutants (Fig. 5). Nevertheless, the impact of BC on *S. pneumoniae* colonisation may explain why exposure to PM is associated with bacterial pneumonia (Brugha and Grigg, 2014; MacIntyre *et al*., 2014; Qiu *et al*., 2014) and exacerbation of chronic infectious diseases such as asthma and COPD (Cortez‐Lugo *et al*., 2015; Deng *et al*., 2015; Xu *et al*., 2016).

This study clearly demonstrates that BC has an intra and inter‐species differential impact on two respiratory pathogens. Therefore BC, and other air pollutants, could also have a significant effect on other bacterial species. The impact of BC on bacteria could affect microbial ecology in many different niches, such as natural microbiomes, resulting in the unbalancing of ecosystems (Fig. 5). In fact, the diversity of marine bacterial communities has been shown to be impacted by BC (Cattaneo *et al*., 2010), an effect that has the potential to cause serious problems for oceanic cycles. Consequently our study shows the importance of the impact of BC on bacteria and the subsequent risks to our health and well‐being.

## Experimental procedures

### Bacterial strains

Methicillin sensitive *Staphylococcus aureus* (MSSA) (SH1000 and Newman), methicillin resistant *Staphylococcus aureus* (MRSA) USA300, and encapsulated (D39) and non‐encapsulated (R6 strain PR201) *S. pneumoniae* isolates were investigated. *S. pneumoniae* strains were cultivated on 5% (v/v) horse blood agar, and *S. aureus* strains cultured on Luria agar (LA). Prior to each assay, overnight cultures were centrifuged, and pellets were resuspended to a matching CFU in the chosen medium.

### Black carbon and quartz

Black Carbon was purchased from Sigma‐Aldrich (UK) under product number 699632. This was provided as a powder with a size distribution of <500 nm, with <500 ppm trace metals, and a weight of 12.01 g/mol. For use in assays, a stock solution of BC was made by dispersing the powder in dH_2_O which had been sterilised by autoclaving at 120˚C at 15 pSI for 15 min. It is noteworthy that BC aggregates in liquid therefore size of particles can change. Quartz reference particles (BCR66 ‐ Distrilab) with a size distribution of 3500–350 nm were also employed in this work. Quartz is chemically stable and therefore does not react with most substances even at high temperatures.

### Biofilm assay

A novel method to elucidate biofilm structure was developed from previous work (Munoz‐Elias *et al*., 2008). This methodology reproducibly quantifies total bacterial growth and gives an indication of the biofilm structure by assessing changes in the ratio of biofilm cells compared with the loosely adherent and planktonic cells. Bacterial cultures (3 ml) were supplemented with 0, 30, 50 or 100 µg/ml black carbon (BC) and seeded into wells of 12‐well plates. Media choice for biofilm formation was determined based on optimisation assays. *S. pneumoniae* D39 biofilms were formed in Todd‐Hewitt Broth + 0.5% (w/v) yeast extract (THY), and PR201 in Brain Heart Infusion (BHI). *S. aureus* SH1000 biofilms were formed in BHI + 4% (w/v) NaCl, and *S. aureus* Newman and USA300 in BHI + 1% (w/v) glucose. 12 well plates were then incubated at 37°C in a 5% (v/v) CO_2_ atmosphere for 24 h. After incubation, planktonic bacteria were gently removed without disrupting the biofilm, and retained. The remaining biofilms were carefully washed with 3 ml phosphate‐buffered saline (PBS) and this wash was also kept. Biofilm bacteria were then removed with CellScrapers (VWR) into 1ml PBS. Each fraction was vortexed for 30 s and serial dilutions were plated to determine colony forming units (CFU).

### Antibiotic tolerance of biofilms

To assess antibiotic sensitivity, the biofilm protocol detailed in the previous section was used. Biofilms were formed in the presence of 100 µg/ml black carbon, and then exposed to antibiotics. For this, the protocol was adjusted so that after the removal of planktonic bacteria and the wash step, either 3 ml medium alone or medium with antibiotics were added to each well and incubated at 37°C 5% (v/v) CO_2_ for 3 h. Biofilms were then washed and CFUs enumerated as for the standard biofilm assay. All antibiotics were used at a concentration which caused significant biofilm degradation but not total biofilm eradication, based on optimisation assays. For *S. aureus*, oxacillin (50 mg/ml in BHI + 2% (w/v) NaCl (Huang *et al*., 1993)), daptomycin (256 µg/ml in BHI + 50 µg/ml CaCl_2_ (Fuchs *et al*., 2002)) and tetracycline (512 µg/ml in BHI) were used. For *S. pneumoniae* PR201, penicillin G (1 µg/ml) was used in BHI.

### Proteolytic degradation of biofilms

To investigate the role of protein in the extracellular biofilm matrix, the standard biofilm protocol previously detailed was again employed. Biofilms were formed in the presence of 100 µg/ml black carbon, and then 3 ml of 100 µg/ml Proteinase K in 10 mM Tris‐ HCl, adjusted to pH 7.5, was added to each well after supernatant removal and washing (Nicholson *et al*., 2013). Control wells containing buffer alone were also included. Biofilms were then incubated at 37°C 5% (v/v) CO2 for 2 h, and bacterial numbers were quantified.

### Microscopy

Bacterial cultures were inoculated into 12‐well plates containing 13 mm round glass coverslips, and were incubated at 37°C in a 5% (v/v) CO_2_ atmosphere for 24 h. For scanning electron microscopy (SEM), coverslips were then washed with PBS, fixed in 2.5% (v/v) glutaraldehyde, washed in 0.1 M Sörensens buffer, gold coated, and viewed with a Hitachi S‐3000H SEM (Dykstra and Reuss, 2003). For transmission electron microscopy (TEM), coverslips were washed with PBS, infused with 10% (v/v) BSA, then fixed with 2.5% (v/v) glutaraldehyde and 1% (w/v) osmium tetroxide/1.5% (w/v) potassium ferricyanide. Coverslips were dehydrated in an ethanol, propylene oxide, Spurr's Modified Resin series and polymerised overnight. Sections of around 90 nm thickness were cut from within each biofilm with a Reichert ultracut E ultramicrotome, stained with 2% (w/v) aqueous uranyl acetate and Reynold's lead citrate, and viewed on a JEOL JEM‐1400 TEM (Glauert and Lewis, 2014). For measurements of biofilm thickness, 18 cross‐sections were taken from the biofilm and were measured blind at 12 equally spaced points with light microscopy.

### *In vivo* colonisation

All experiments were carried out in accordance with the Home Office Project Licence and adhered to the UK Animals (Scientific Procedures) Act (1986). Naïve 8 week old female outbred MF1 mice were obtained from Charles River, UK. Upon arrival, mice were housed in groups of 2–5, depending on study requirements, and allowed to acclimatise for one week in the Division of Biomedical Services, University of Leicester, on a 12 h light‐dark cycle and access to food and water *ad libitum*. For the colonisation experiment, mice were intranasally inoculated with 15 µl of either (i) PBS as a control, (ii) 6.45 × 10^5^ CFU *S. pneumoniae* D39, (iii) 7 mg/ml BC alone, or (iv) a mixture of 6.45x10^5^ CFU *S. pneumoniae* D39 with 7 mg/ml BC, whilst held in a horizontal position. For inoculation, mice were anaesthetised with 2.5% (v/v) isoflurane and held in a horizontal position during inoculation. Seven mice were used per group, 28 in total. Allocation of mice to each group, the order in which groups were inoculated, and the order in which each cage was culled for sample recovery, was randomised. Mice were regularly monitored (at least once daily) for signs of disease for the duration of the experiment. At 7 days post‐infection, five mice were culled from each group to assess bacterial load. Mice were deeply anaesthetised with 2.5% (v/v) isoflorane and blood was collected by cardiac puncture in which the animal was exsanguinated under terminal anaesthesia. Death was confirmed by cervical dislocation. Bronchoalveolar and nasopharyngeal lavages were performed to determine bacterial carriage at each time‐point. Bronchoalveolar lavages were performed by instilling 500 µl tripticase soy broth supplemented with 10% (v/v) glycerol (TSBG) into the lungs via the trachea, recovering the lavage fluid, and repeating this twice. Nasopharyngeal lavages were performed by washing through the nasopharynx with 500 µl TSBG three times. Lavages were then serially diluted and plated to determine bacterial viability. For histological analysis, an additional two mice per group were deeply anaesthetised with 2.5% (v/v) isoflurane and culled by cervical dislocation. Death was confirmed by exsanguination. The lungs, spleen, cervical lymph nodes and nasopharyngeal tissue were then removed and fixed in 10% (v/v) formalin for 24–48 h. Samples were embedded in paraffin for histological sectioning, and were stained with haematoxylin and eosin and analysed by light microscopy.

### Statistical analysis

Data are summarised as standard error of the mean (SEM) and were analysed using GraphPad Prism version 6.04 (GraphPad Software Inc., La Jolla, CA). Biofilm quantification results were validated by at least three biological replicate experiments. *In vivo* experiments were carried out with *n* = 7 in each group. Quantification of biofilm thickness was carried out on *n* = 18 distinct biofilm sections per condition. ANOVA or t‐tests were used to determine significance as appropriate.

## Supporting information

Additional Supporting Information may be found in the online version of this article at the publisher's website:

**Fig. S1.** The effect of black carbon on internal biofilm structure. Biofilms of *S. pneumoniae* (a,b), *S. aureus* SH1000 (c,d) and Newman (e,f) were cultured in the presence or absence of 100 μg/ml BC and imaged by transmission electron microscopy (TEM). Images are representative of the entire biofilm structure.
**Fig. S2.** Quartz has no effect on biofilm architecture. Biofilms of *S. aureus* SH1000 (a, b) and *S. pneumoniae* PR201 (c,d) were cultured with and without 30 μg/ml Quartz and imaged by scanning electron microscopy (SEM). Images are representative of the entire biofilm structure.
**Fig. S3.** The effect of black carbon on *S. aureus* Newman biofilms. Biofilms of *S. aureus* Newman were cultured in the presence or absence of 30 ‐ 100 μg/ml BC. Biofilms were imaged by scanning electron microscopy (SEM) at increasing resolution (a‐d) and light microscopy was used to quantify biofilm thickness (e‐g, n=18). Viable bacterial cells were measured by sequential removal and quantification of planktonic, loosely‐ adhered, and biofilm bacteria (h, n=4). Error bars represent ± 1 SEM. Significance was determined by t‐tests (g) or ANOVA (h) * p≤0.05, *** p≤0.001, **** p≤0.0001.
**Fig. S4.** The effect of black carbon on *S. aureus* USA300 biofilms. Biofilms of *S. aureus* USA300 were cultured in the presence or absence of 30 ‐ 100 μg/ml BC. Biofilms were imaged by scanning electron microscopy at increasing resolution (a‐d). Images are representative of the entire biofilm structure. Viable bacterial cells were measured by sequential removal and quantification of planktonic, loosely‐ adhered, and biofilm bacteria (e, n=4). Error bars represent ± 1 SEM. Significance was determined by ANOVA.
**Fig. S5.** BC alters biofilm antibiotic tolerance. Biofilms of *S. aureus* SH1000 (a), Newman (b), and USA300 (c) were cultured in the presence or absence of 100 μg/ml BC for 24 h. Planktonic and loosely‐adhered bacteria were then removed and biofilms were either incubated with 3 ml of 50 mg/ml oxacillin in PBS supplemented with 2% (w/v) NaCl, or with buffer alone as a control, for 3 h at 37°C in 5% (v/v) CO_2_. After incubation, planktonic and loosely adherent bacteria were removed and discarded, and the remaining biofilm bacteria were quantified. For *S. aureus*, oxacillin (50 mg/ml in BHI + 2% (w/v) NaCl), daptomycin (256 µg/ml in BHI + 50 µg/ml CaCl_2_) and tetracycline (512 µg/ml in BHI) were used. For *S. pneumoniae* PR201, penicillin G (1 µg/ml) was used in BHI. Planktonic and loosely‐adhered cells were then removed and biofilm bacteria quantified. n≥3. Error bars represent ± 1 SEM. Significance was determined by unpaired t‐tests. * p≤0.05, ** p≤0.01.
**Fig. S6.** Histological analysis of lungs and nares at 7 days post‐inoculation. Female MF1 mice were mice intranasally inoclated with 15 µl of PBS, Black Carbon (BC), *S. pneumoniae* D39 (D39), or D39 and BC together (D39+BC). Mice were culled 7 days post inoculation. Formalin fixed, paraffin embedded tissue sections of lungs (A‐D) and nares (E‐H) were stained with haematoxylin and eosin (H+E). Sections were imaged using light microscopy using 5x or 10x magnification. Physical features of notes are labelled for identification.
**Fig. S7.** Histological analysis of cervical lymph nodes and spleen at 7 days post‐inoculation. Female MF1 mice were mice intranasally inoculated with 15 µl of PBS, Black Carbon (BC), *S. pneumoniae* D39 (D39), or D39 and BC together (D39+BC). Mice were culled 7 days post inoculation. Formalin fixed, paraffin embedded tissue sections of cervical lymph nodes (A‐D) and spleen (E‐H) were stained with haematoxylin and eosin (H+E). Sections were imaged using light microscopy using 5x or 10x magnification. Physical features of notes are labelled for identification. Lymph nodes: HEV ‐ High Endothelial Venules, the entry point for lymphocytes into lymph nodes. Spleen: PALS ‐ Peripheral Arteriolar Lymphoid Sheath. Follicles comprised of a germinal centre (GC), a mantle (Mn) zone, and a marginal (Mg) zone. White pulp comprises PALS, follicles, and lymphoid cells, surrounded by Red Pulp (RP).
