# Supplementary material for: Air pollution alters Staphylococcus aureus and Streptococcus pneumoniae biofilms, antibiotic tolerance and colonisation
Source: Environ Microbiol. 2017 Feb 28;19(5):1868–80. doi: 10.1111/1462-2920.13686 (PMC6849702; doi:10.1111/1462-2920.13686)
Supplement: Supplementary file 1 — Fig. S1. The effect of black carbon on internal biofilm structure. Biofilms of S. pneumoniae (a,b), S. aureus SH1000 (c,d) and Newman (e,f) were cultured in the presence or absence of 100 μg/ml BC and imaged by transmission electron microscopy (TEM). Images are representative of the entire biofilm structure. Fig. S2. Quartz has no effect on biofilm architecture. Biofilms of S. aureus SH1000 (a, b) and S. pneumoniae PR201 (c,d) were cultured with and without 30 μg/ml Quartz and imaged by scanning electron microscopy (SEM). Images are representative of the entire biofilm structure. Fig. S3. The effect of black carbon on S. aureus Newman biofilms. Biofilms of S. aureus Newman were cultured in the presence or absence of 30 ‐ 100 μg/ml BC. Biofilms were imaged by scanning electron microscopy (SEM) at increasing resolution (a‐d) and light microscopy was used to quantify biofilm thickness (e‐g, n=18). Viable bacterial cells were measured by sequential removal and quantification of planktonic, loosely‐ adhered, and biofilm bacteria (h, n=4). Error bars represent ± 1 SEM. Significance was determined by t‐tests (g) or ANOVA (h) * p≤0.05, *** p≤0.001, **** p≤0.0001. Fig. S4. The effect of black carbon on S. aureus USA300 biofilms. Biofilms of S. aureus USA300 were cultured in the presence or absence of 30 ‐ 100 μg/ml BC. Biofilms were imaged by scanning electron microscopy at increasing resolution (a‐d). Images are representative of the entire biofilm structure. Viable bacterial cells were measured by sequential removal and quantification of planktonic, loosely‐ adhered, and biofilm bacteria (e, n=4). Error bars represent ± 1 SEM. Significance was determined by ANOVA. Fig. S5. BC alters biofilm antibiotic tolerance. Biofilms of S. aureus SH1000 (a), Newman (b), and USA300 (c) were cultured in the presence or absence of 100 μg/ml BC for 24 h. Planktonic and loosely‐adhered bacteria were then removed and biofilms were either incubated with 3 ml of 50 mg/ml oxacillin in PBS suppl [file EMI-19-1868-s001.pptx]

## Slide 1
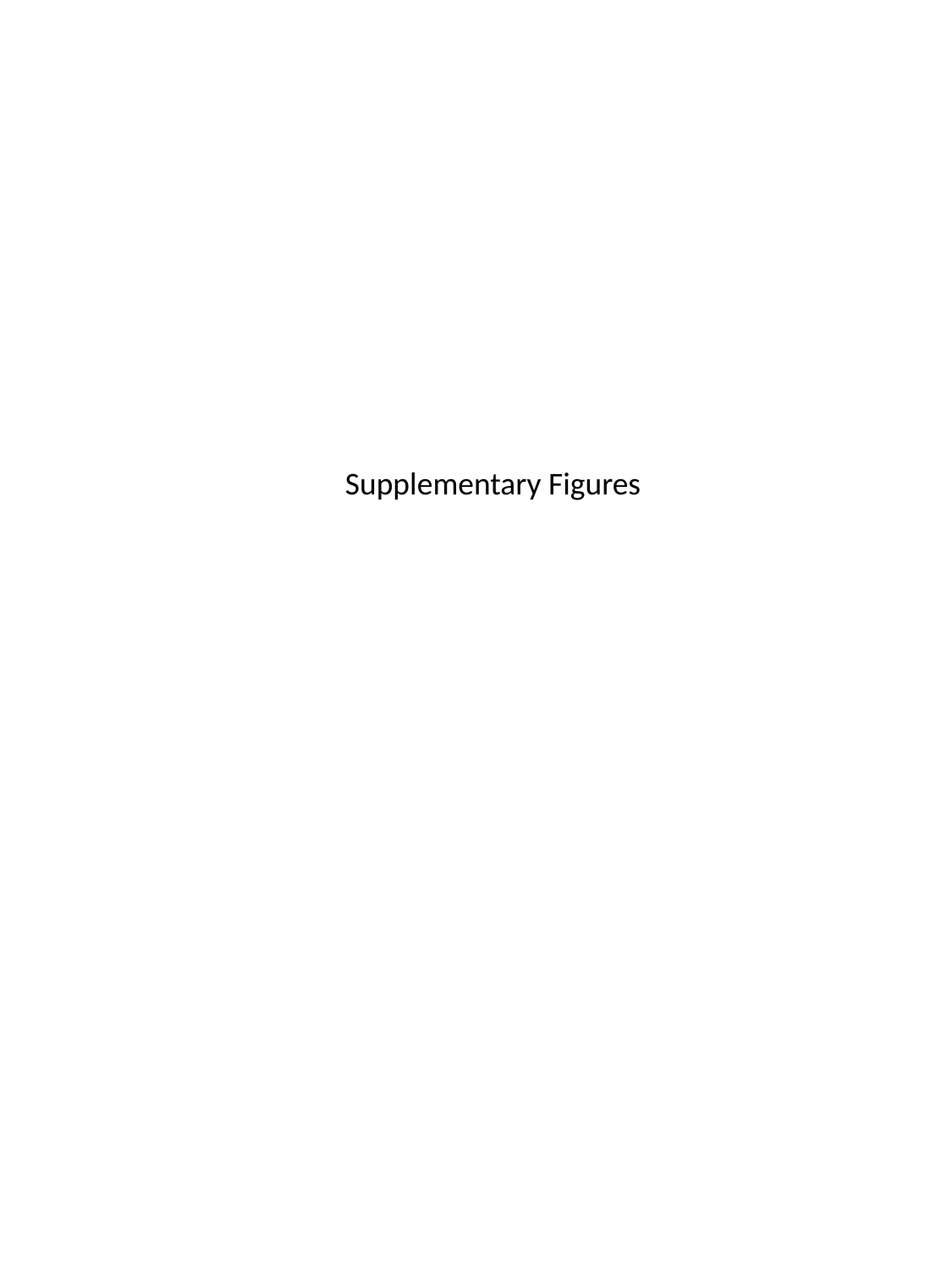

Supplementary Figures

## Slide 2
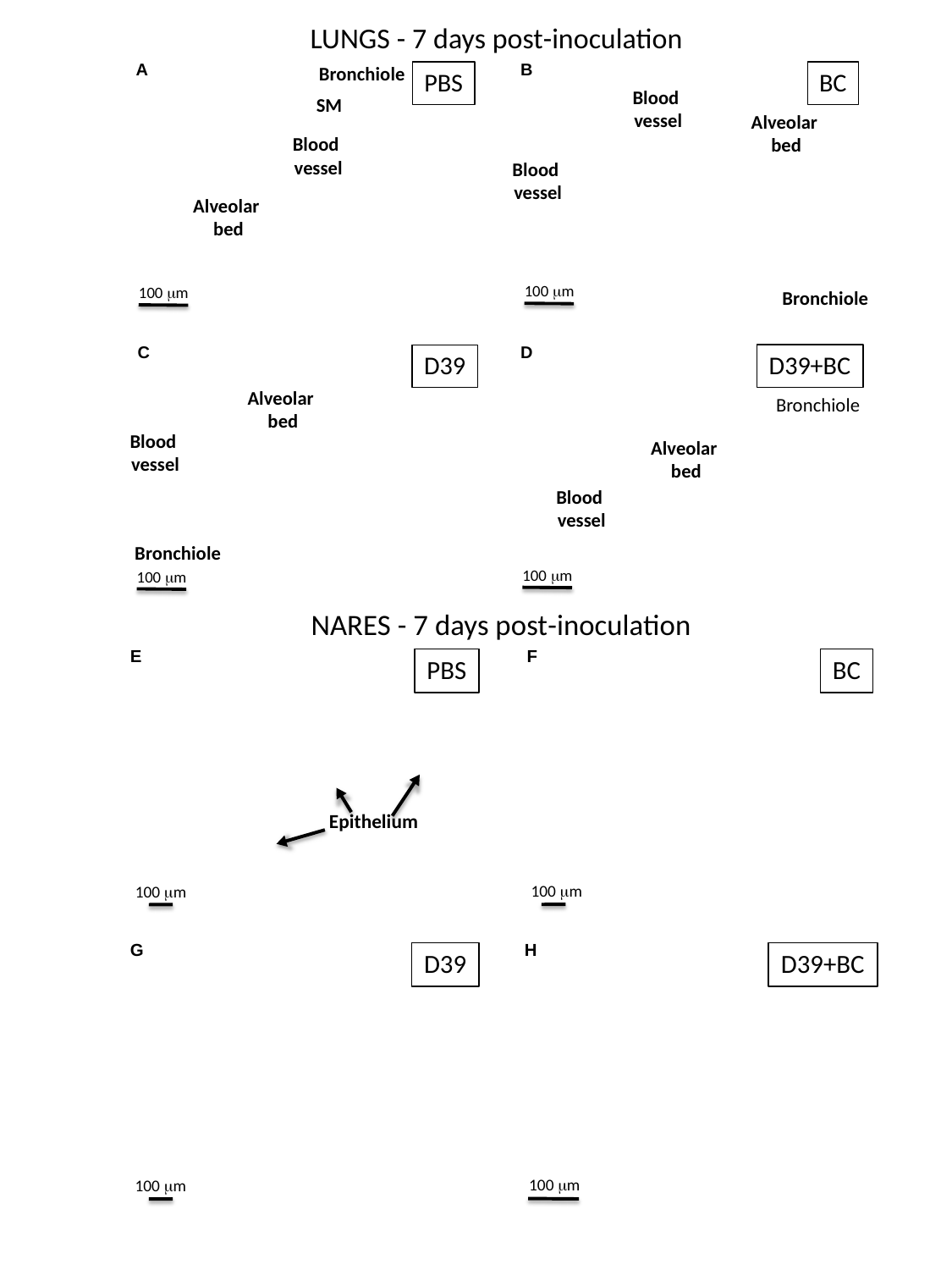

## Slide 3
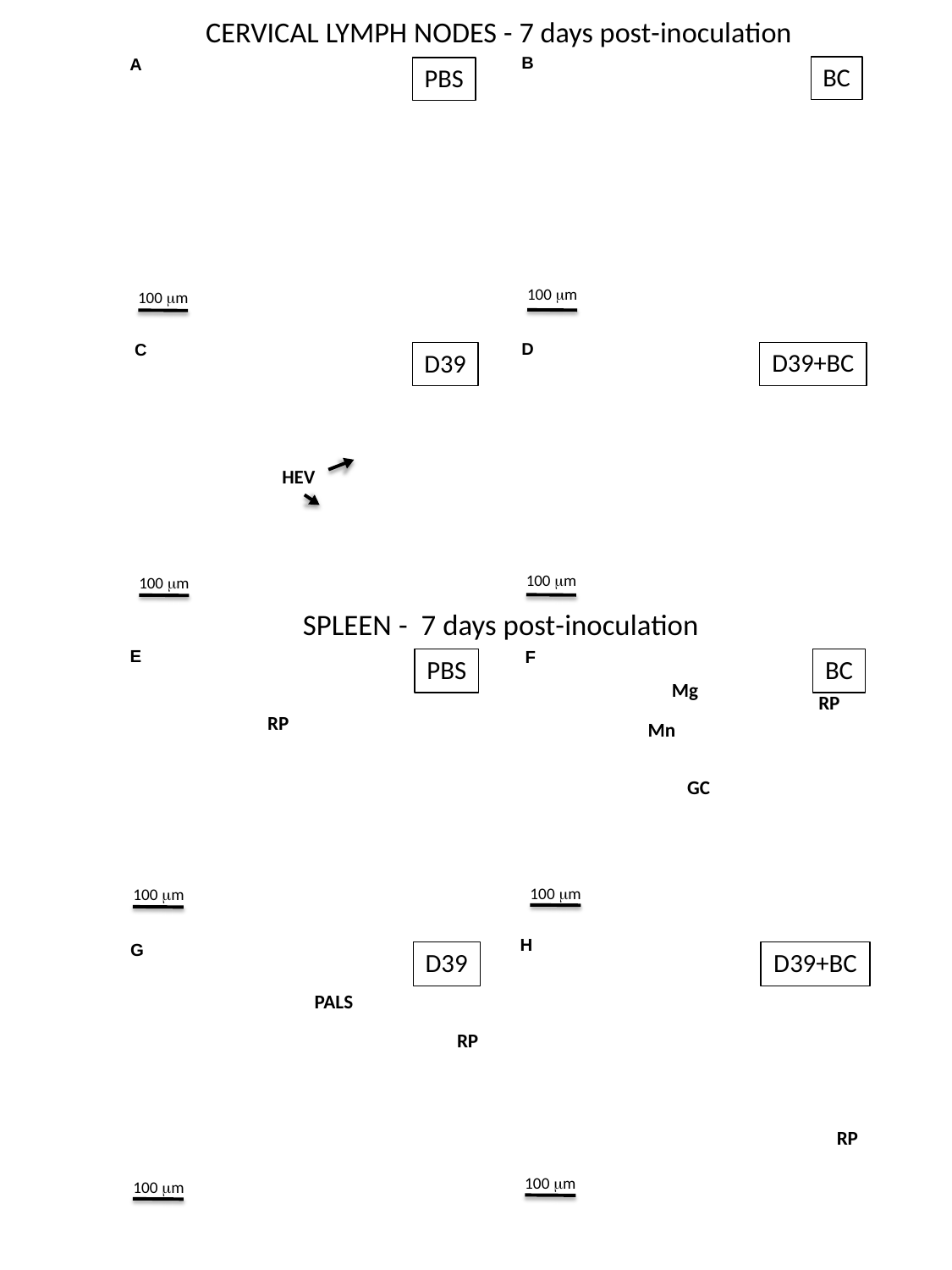

## Slide 4
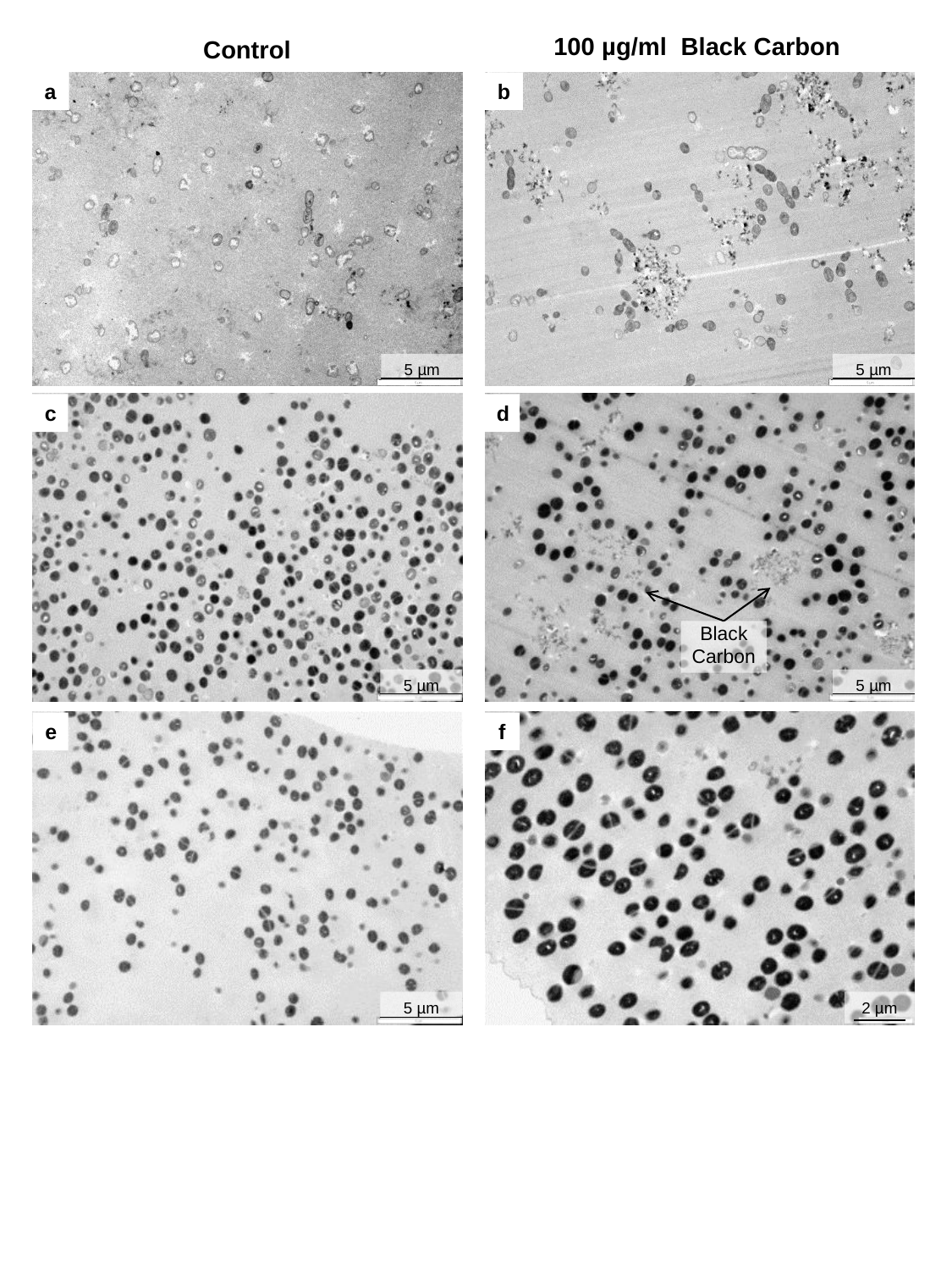

100 µg/ml Black Carbon
Control
a
b
b
a
5 µm
5 µm
c
d
Black
Carbon
5 µm
5 µm
f
e
2 µm
5 µm

## Slide 5
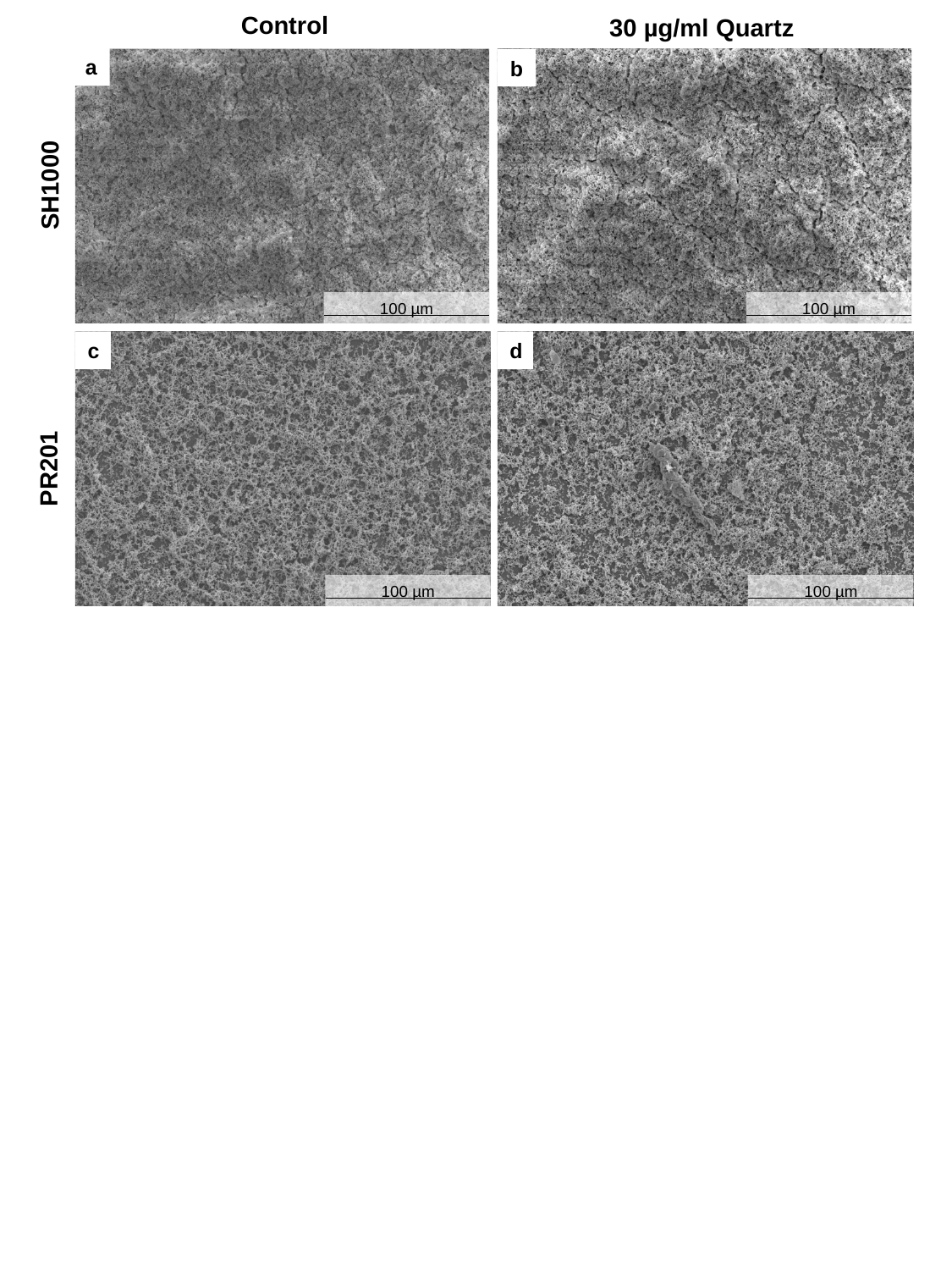

Control
30 µg/ml Quartz
a
b
SH1000
100 µm
100 µm
c
d
PR201
100 µm
100 µm

## Slide 6
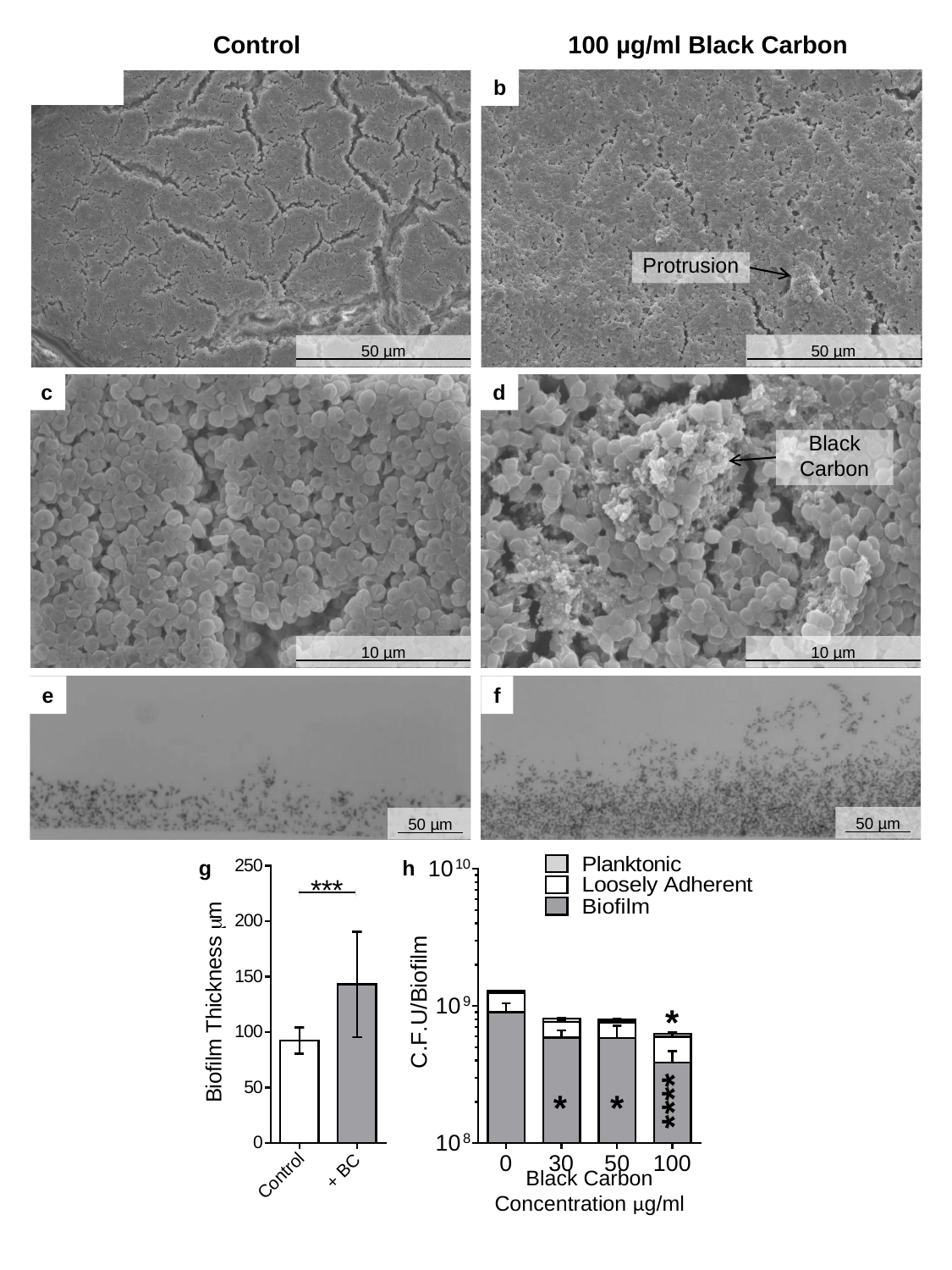

Control
100 µg/ml Black Carbon
a
b
Protrusion
50 µm
50 µm
c
d
Black Carbon
10 µm
10 µm
e
f
50 µm
50 µm
h
g
*
****
*
*
Black Carbon
Concentration μg/ml

## Slide 7
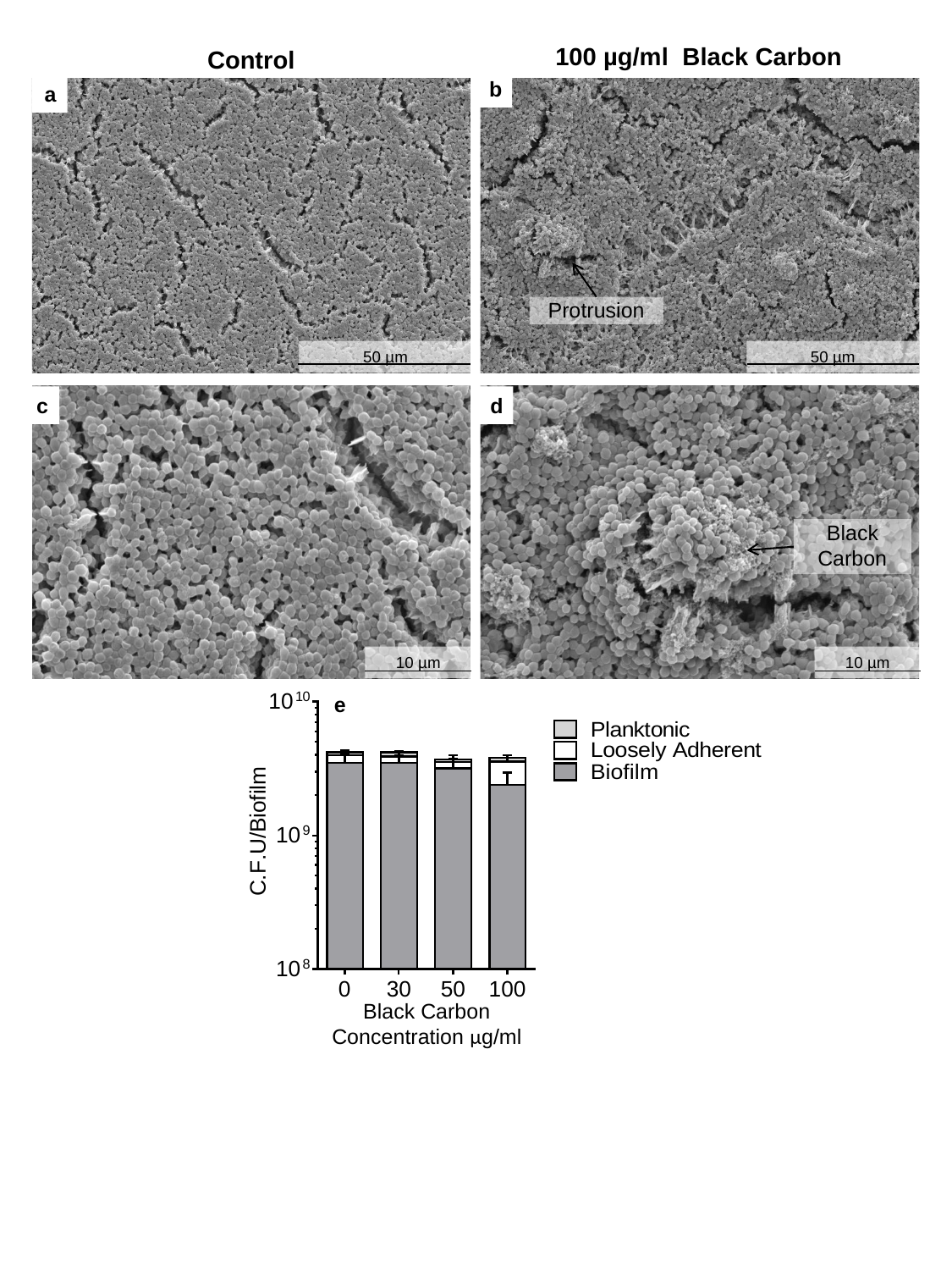

100 µg/ml Black Carbon
Control
b
a
Protrusion
50 µm
50 µm
c
d
Black Carbon
10 µm
10 µm
e
Black Carbon
Concentration μg/ml

## Slide 8
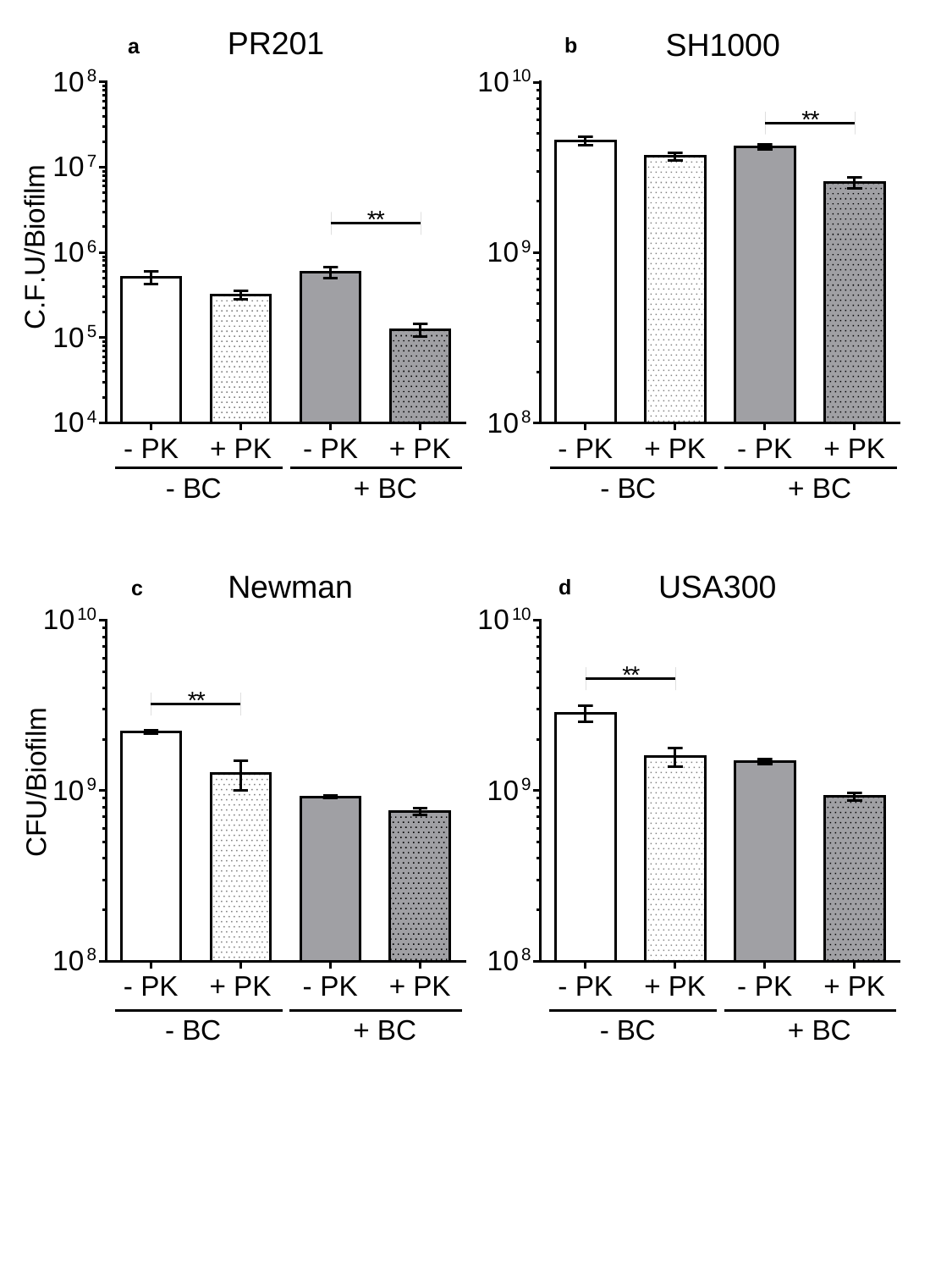

PR201
SH1000
b
a
Newman
USA300
d
c

## Slide 9
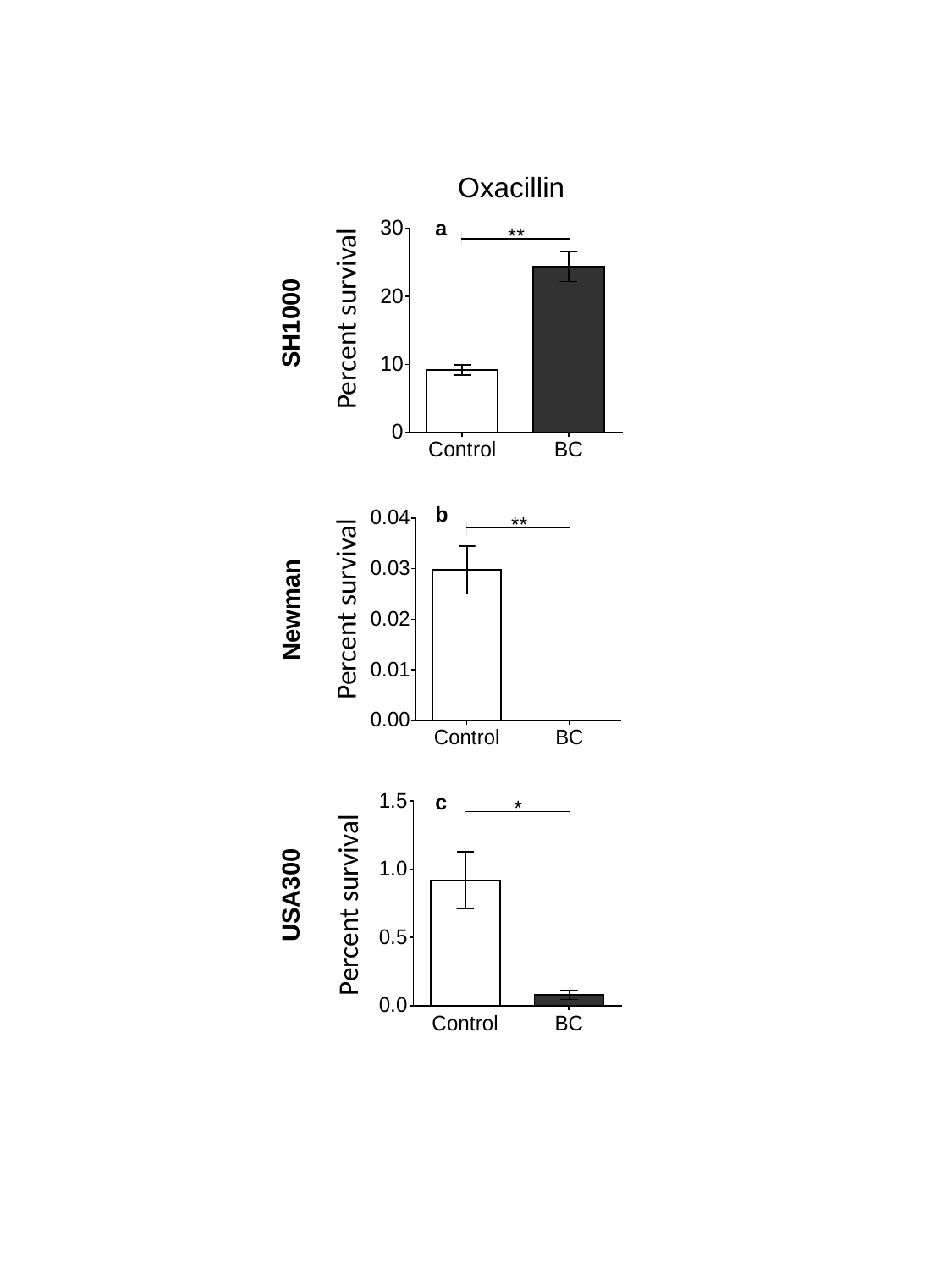

Oxacillin
a
Percent survival
SH1000
b
Percent survival
Newman
c
USA300
Percent survival
